# Palm Fruit Bioactive Complex (PFBc), a Source of Polyphenols, Demonstrates Potential Benefits for Inflammaging and Related Cognitive Function

**DOI:** 10.3390/nu13041127

**Published:** 2021-03-30

**Authors:** Susan J. Hewlings, Kristin Draayer, Douglas S. Kalman

**Affiliations:** 1 The Herbert H & Grace A. Dow College of Health Professions, Nutrition, Central Michigan University, Mt. Pleasant, MI 48859, USA; 2 Nutrasource/GRAS Associates, Scientific Affairs, Guelph, ON N1G0B4, Canada; dkalman@nova.edu; 3 EDGE Veterinary Vaccines Consulting Group, 315 MAIN STREET 201, Ames, IA 50010, USA; kedraayer@gmail.com; 4 Nutrion Department, College of Osteopathic Medicine, Nova Southeastern University, Fort Lauderdale, FL 33314, USA

**Keywords:** Palm Fruit Bioactive complex, PFBc, inflammaging, mild cognitive impairment, polyphenols, antioxidant, cognitive function, anti-inflammatory

## Abstract

Cognitive function is a key aspect of healthy aging. Inflammation associated with normal aging, also called inflammaging is a primary risk factor for cognitive decline. A diet high in fruits and vegetable and lower in calories, particularly a Mediterranean Diet, may lower the risk of age-related cognitive decline due in part to the associated high intake of antioxidants and polyphenols. A phenolic, Palm Fruit Bioactive complex (PFBc) derived from the extraction process of palm oil from oil palm fruit (*Elaeis guineensis*), is reported to offset inflammation due to its high antioxidant, especially vitamin E, and polyphenol content. The benefit is thought to be achieved via the influence of antioxidants on gene expression. It is the purpose of this comprehensive review to discuss the etiology, including gene expression, of mild cognitive impairment (MCI) specific to dietary intake of antioxidants and polyphenols and to focus on the potential impact of nutritional interventions specifically PFBc has on MCI. Several in vitro, in vivo and animal studies support multiple benefits of PFBc especially for improving cognitive function via anti-inflammatory and antioxidant mechanisms. While more human studies are needed, those completed thus far support the benefit of consuming PFBc to enhance cognitive function via its anti-inflammatory antioxidant functions.

## 1. Introduction

As the world’s aged population expands, defining “healthy aging” and the steps for achieving it have become a significant focus of preventative health research. While there are many definitions and concepts suggested as part of “healthy aging” one that incorporates all aspects of wellness seems most appropriate and in keeping with modern concepts of overall health and wellness. Kuh et al. defined healthy aging in a succinct yet comprehensive way using three principles: 1. “survival to old age”; 2. “delay in the onset of chronic diseases and disabilities”; and 3. “optimal functioning for the maximal time period” [1]. The World Health Organization defined “healthy aging” as “the process of developing and maintaining functional ability that enables well-being in older age” [2]. Both statements expand upon these definitions to include social functioning, societal acceptance and related sociological aspects, highlighting the multidimensional aspects of wellness in aging and recognizing that it is more than just surviving. In addition, both sets of definitions recognize the importance of mental health and discuss its role in reference to all other dimensions associated with “healthy aging”.

In the context of such comprehensive definitions, health behaviors and interventions to achieve healthy aging must then also be discussed from a multidimensional wellness perspective and include the physical, behavioral and social dimensions of health. It could then be argued that cognitive function might be at the center of these dimensions and to focus on achieving and maintaining it would impact the other dimensions of healthy aging. This also makes sense when one considers that cognitive decline has been shown to negatively impact mortality [3]. While some epidemiological studies have suggested a slight decline in incidence of dementia over the last three decade in the United States, this may not be reflective of the global incidence [4]. Furthermore, these data may not be reflective of incidence of cognitive decline or impairment which is associated with more subjective measures and therefore becomes more challenging to define and measure. The term “Mild cognitive impairment (MCI)” was introduced into the literature by Reisberg and colleagues and was generally used to refer to a period of transition between normal cognitive function and clinically diagnosed Alzheimer’s disease (AD) [5]. The Diagnostic and Statistical Manual of Mental Disorders 5th Edition (DSM-V) classifies MCI as a “mild neurocognitive disorder.” It states that there must be both a subjective and objective decline in one or more of the six cognitive domains, not substantially interfering with instrumental activities of daily living, and not occurring in the context of delirium or other psychological disorders [6,7]. MCI occurs along a continuum from normal cognition to dementia and while not all individuals with MCI progress to AD, MCI alone can decrease quality of life and increase risk of other negative health outcomes [8]. Incidence has been reported to range from a low of 3% to a high of 22% in individuals over 65 years of age [9]. One study found mild cognitive decline in skills starts in the late 20′s (between 22 and 27 years of age) [10]. These facts may allow for a greater chance of intervention to prolong age of onset, prevent or minimize the chances of lifetime cognitive impairment. Therefore, despite challenges in defining and measuring MCI, identifying its potential etiology and thereby establishing appropriate prevention strategies and interventions targeting associated mechanisms is critical.

One such mechanism associated with the inflammation commonly seen in aging is called “inflammaging” [11]. The risk factors identified for MCI are thought to act via inflammaging and other mechanisms. These risk factors include family history, the presence of vascular risk factors such as hypertension, hyperlipidemia, coronary artery disease, and stroke [12], and having the apolipoprotein E allele [13]. Age is the strongest risk factor, with a diet low in fruits and vegetables and polyphenols and lack of physical activity (lifestyle) also recognized as strong contributing factors [14,15]. A diet rich in fruits and vegetables has been shown to lower the risk of chronic diseases including age-related cognitive decline and the risk of developing neurodegenerative disease [16,17,18]. This benefit is thought to be mediated by the higher concentration of a variety of polyphenols, along with antioxidants that naturally occur in fruits and vegetables and other foodstuffs, and by such a dietary pattern that in turn may help to offset the negative health outcomes associated with inflammaging [19,20]. The benefits of polyphenols may occur via their impact on gene expression. One such phenolic (polyphenol) that has been shown to influence gene expression is Palm Fruit Bioactive complex (PFBc), which is derived from the extraction process of palm oil from oil palm fruit (*Elaeis guineensis*). Palm oil has been used for many therapeutic effects most attributed to its high antioxidant and polyphenol content [21]. It is the purpose of this comprehensive review to discuss the etiology, including gene expression, of MCI specific to dietary intake of antioxidants and polyphenols and to focus on the impact of nutritional interventions specifically PFBc. A search of the literature was conducted using Pubmed.gov and Google Scholar databases with the search terms polyphenols or phytochemicals or palm fruit or *Elaeis guineensis* and inflammaging or anti-aging or mild cognitive impairment.

## 2. Etiology of MCI

Risk factors identified for AD include low education, polypharmacy, physical inactivity, smoking, depression, diabetes, midlife hypertension and midlife obesity, accounting for about a third of cases worldwide [9,14]. Second to aging, physical inactivity has been suggested to be the greatest risk factor [14]. Whether risk factors are the same for MCI is not clear. A recent study analyzed data obtained from the 2016 Centers for Disease Control and Prevention (CDC) based on Behavioral Risk Factor Surveillance System (BRFSS) nationwide telephone survey. They reported that the factors contributing to MCI include depression, physical health, cigarette usage, education level, sleep time, kidney disease, alcohol consumption, and exercise [15]. It has been identified that there are several reversible risk factors for MCI that should be focused on as addressing these factors may help to delay or offset the progression of MCI to dementia. Polypharmacy, depression, high blood pressure, hypoglycemia, hyperglycemia, vitamin B12 deficiency, dehydration and lack of physical activity and dietary pattern have all been identified as reversible factors [9]. The Mediterranean Diet has been shown to be beneficial, most likely due to a higher intake of antioxidants and polyphenols [22]. A meta-analysis of prospective studies reported a strong association between physical activity and cognitive decline whereby those who exercise are 38% less likely to experience MCI than those who do not exercise [23]. The exact mechanisms that lead to the benefit are not clear but it is thought that exercise increases endogenous antioxidants [24], increases release of brain derived neurotrophic factor (BDNF), stimulates blood flow to the brain, protects against lifestyle diseases, reduces stress and cortisol levels, and stimulates the release of neurotrophins, associated with optimal neuronal health [25]. Assessing the mechanisms associated with age related physiological changes may provide insight as to the mechanisms associated with MCI.

### 2.1. Inflammation/Inflammaging

While there are many obvious and well-studied aspects of aging such as sarcopenia, frailty, and MCI, the mechanisms driving these aging related challenges to physiological homeostasis are less understood. It is thought to be a complex interplay of changes that impact tissue and thus systemic function, and it is hypothesized that inflammation is an important driver for age related physiological changes. It is recognized that the aging process is associated with activation of inflammatory pathways, a phenomenon known as “inflammaging” [11]. “Inflammaging” describes the low-grade, chronic, systemic inflammation in aging, in the absence of infection and is a risk factor for morbidity and mortality in aged people [19]. While several mechanisms may drive the process, the mitochondria play a major role in inflammaging and in the activation of Nlrp3 inflammasome. The Nlrp3 inflammasome is a multiprotein complex that can activate pro-caspase-1 activity in response to cellular challenges resulting in the processing and secretion of the proinflammatory cytokines IL-1β and IL-18. Most activators of the Nlrp3 inflammasome induce the generation of mitochondrial reactive oxygen species (ROS, free radicals) [19], thus suggesting an increased need for antioxidants and anti-inflammatory compounds in ageing individuals to prevent the impact of inflammaging before it occurs. It is always desirable to explore dietary sources as a primary means for enhancing nutrient intake; therefore, nutritional interventions have been investigated as to their impact on inflammaging.

Though inflammation is still at the center of research related to etiology, the primary theory regarding the development of neurodegenerative disease has shifted from a focus on neurons to astrocytes [26]. Astrocytes are glial cells found in the central nervous system and when they are activated they stimulate the production of cytokines and other inflammatory mediators [27]. In neurodegenerative diseases the signaling pathways for cytokines and chemokines become dysregulated [28]. In vitro interleukin IL-1B activates astrocytes [29]. The activated glial cells also generate ROSand reactive nitrogen species (RNS) leading to oxidative damage, which also plays a role in neurodegenerative disease [30,31]. Polyphenols, such as PFBc have antioxidant and anti-inflammatory functions and therefore may offer some protection [32,33] An in vitro study of IL-1B-activated astrocytes showed the release of multiple inflammatory mediators that were inhibited by the presence of PFBc. PFBc reduced the release of pro-inflammatory cytokines and chemokines including tumor necrosis factor alpha (TNFα), regulated upon activation, normal T cell expressed and presumably secreted (RANTES) and IP-10; PFBc reduced ROS by detoxifying oxygen free radicals and increasing superoxide dismutase (SOD-1); and PFBc modulated the expression of the cell-surface adhesion molecules intercellular adhesion molecule (ICAM) and vascular cell adhesion molecule (VCAM) associated with inflammation. These results suggest that PFBc may have health and beneficial health effects for those with neuroinflammation and inflammation associated with neurodegenerative diseases [34].

### 2.2. Gene Expression and Brain Health

Apolipoprotein E (APOE), Amyloid Beta Precursor Protein (APP), and Presenilin-1 (PSEN1) are well-known genes associated with both MCI and AD. APOE, especially ϵ4 allele, is the most statistically associated gene to MCI [35]. Recent research has shown that dysregulated regulatory elements, such as microRNAs (miRNAs)–gene interactions may lead to epigenetic alterations involved with cognitive and neurodegeneration processes such that there is a detectable difference between those with MCI, AD and those who are healthy [36]. The miRNAs may serve as a biomarker and a target for intervention [37]. Prooxidants play an important role in aging and chronic diseases and they also regulate gene expression [38], particularly the transcription factors, nuclear factor kappa-B and activator protein-1 which in turn trigger inflammation [39]. Therefore, compounds that inhibit prooxidants, such as antioxidants may in turn prevent or reduce inflammation and thereby help to mitigate chronic diseases associated with inflammation at the gene level, including MCI. Furthermore, some antioxidants, such as phenolics are known to influence gene expression [40]. For example, it has been reported that phenolic compounds modulate the expression of cyclooxygenase-2 (COX-2) [41] through suppression of activation of nuclear factor activator protein-1 (AP-1) [42] to increase the expression of antioxidant enzymes [43]. Therefore, a diet or supplemental protocol high in antioxidants, particularly phenolics may help to mitigate chronic disease by decreasing inflammation and may do so at the genetic level.

## 3. Nutritional Interventions

Research suggests that a diet rich in antioxidants (such as fruits, nuts, vegetables, and spices) and thus higher in polyphenols and lower in calories, may lower the risk of age-related cognitive decline, and therefore the risk of developing neurodegenerative disease [16,17,18]. In addition, research supports the role of B-vitamins (vitamin B12, vitamin B6, riboflavin), vitamin D, folate, and nutrients such as polyphenols, and omega-3 polyunsaturated fatty acids (PUFAs)- eicosapentaenoic acid (EPA) and docosahexaenoic acid (DHA) in neuroprotection and in reducing the risk of cognitive decline [44]. In support of the benefits of such a dietary pattern, recent systematic reviews have reported a strong association between the Mediterranean-style diet (MsD) and neurodegenerative conditions [45,46,47] as well as type 2 diabetes and cardiovascular diseases [48].

The MsD is an eating pattern based on the countries bordering the Mediterranean Sea such as Greece and Italy. However, there is no agreed upon definition of the MsD. It is not a specific diet but rather a collection of eating patterns from various countries in the Mediterranean region. Diets of different countries around the Mediterranean Sea vary from one another slightly but do share some similar dietary patterns characterized by plenty of plant foods, olive oil as the primary fat, moderate amounts of dairy, fish, poultry and wine and low consumption of red meat., Oldways Preservation and Exchange Trust in collaboration with the European Office of the World Health Organization and Harvard School of Public Health introduced the Mediterranean Diet Pyramid to try and define characteristics of this dietary pattern emphasizing vegetables, fruits, whole grains, herbs and spices, olive oil, beans and nuts, moderate amounts of fish, seafood, dairy and poultry and limited amounts of red meats and sweets [49].

In a meta-analysis of twelve prospective cohort studies, adherence to a MsDwas analyzed in relation to mortality and incidence of chronic diseases. It was found that greater adherence to a MsD was associated with a significant reduction in the incidence of AD [50]. An update to this systematic review and meta-analysis including 7 more prospective cohort studies confirmed the results of the previous meta-analysis with an extension of the findings to include mild cognitive impairment as well [51]. In a systematic review assessing the association between adherence to a MsDand cognitive function and dementia, including 11 observational studies and 1 randomized controlled trial, it was found that adherence to a MsD was associated with reduced risk of AD, better cognitive function, and lower rates of cognitive decline in 9 out of the 12 studies but results on mild cognitive impairment were inconsistent [52]. A more recent systematic review of the effects of MsD on cognitive function, cognitive impairment, all-type dementia, and AD included 32 studies from 25 unique cohorts (27 observational studies and 5 RCTs). It was found that adherence to a MsD was associated with better cognitive performance [53]. In a comprehensive umbrella review of meta-analyses of prospective studies on dietary factors and neurodegenerative disorders, the MsD was inversely associated with neurodegenerative disorders, but the quality of evidence was generally low [54]. While the results of these studies are promising, more long-term prospective cohort studies beginning in early to mid-life and more experimental data from randomized controlled trials are needed for conclusive results on the effects of the MsD on cognitive function.

The MIND (Mediterranean-DASH Intervention for Neurodegenerative Delay) diet has been studied in relation to cognitive decline with promising results. The MIND diet, as the name implies, is a hybrid of the Mediterranean diet and Dietary Approaches to Stop Hypertension the (DASH) diet. These diets have previously shown to lower risk of cardiovascular diseases. Researchers at Rush University Medical Center and Harvard School of Public Health merged these two dietary patterns with modifications based on ease of following the diet and on research on diet and dementia to come up with this hypothesis-based dietary pattern [55,56]. There are 15 dietary components of the MIND diet, 10 brain-healthy food groups and 5 unhealthy food groups. The 10 food groups in the brain-healthy category include leafy green vegetables, other vegetables, berries, olive oil, fish, poultry, nuts, beans, whole grains, and wine. The 5 food groups in the unhealthy category include red meats, cheese, butter, fried foods and pastries/sweets. It is recommended to eat at least 3 servings of whole grains, 1 salad and 1 other vegetable in addition to a salad and 1 glass of wine daily with olive oil used as the main fat. Berries and poultry are recommended to be consumed at least twice per week, fish at least once per week, beans every other day and 5 servings of nuts per week. Red meat is limited to 4 times per week, pastries to 5 times per week, and butter, cheese and fried/fast food to no more than 1 time per week [55,56].

In the original research on the MIND diet, a MIND diet score was devised based on dietary components found to be neuroprotective. Change in cognition was assessed in 960 participants of the Memory and Aging Project (MAP) over an average of 4.7 years. This study found that the MIND score was positively associated with slower cognitive decline [55]. In a later study assessing the MIND diet, the MsD, and the DASH diet in relation to incidence ofAD, it was found that high and moderate adherence to the MIND diet was associated with lower rates of AD versus the lowest adherence rates to the diet whereas only high adherence of DASH and the MsD was associated with lower rates of AD versus moderate and low adherence to the diets [57].

More recently, a systematic review was conducted examining the association of the MsD, the DASH diet and the MIND diet with AD, dementia, and cognitive decline. This review found that higher adherence to all three diets was associated with lower risk of AD and less cognitive decline with the MIND diet having the strongest associations [58]. Another systematic review assessing dietary patterns and cognitive health in older adults found that plant-based dietary patterns rich in mono- and PUFAs and lower in processed food consumption including the MIND, DASH, MsD, and anti-inflammatory diet, were associated with positive effects on cognitive health outcomes in older adults [59].

This association between the MsD and other plant based diets and reduction in cognitive decline is thought to be due to the antioxidant and anti-inflammatory effects of the high fruit and vegetable intake [20]. Similarly, when individual nutrients such as n-3 fatty acids, DHA, flavonoids and polyphenols show benefits for preventing cognitive decline the mechanism of action by which they exert their effect is thought to be via antioxidant and/or anti-inflammatory pathways [60,61]. Among these individual nutrients, polyphenols have perhaps received the most attention.

### 3.1. Polyphenols

Polyphenols are a complex group of non-nutritive compounds found in plant foods including fruits and vegetables, herbs and spices, chocolate, tea, coffee, whole grains and red wine. Though they are thought to be non-essential in the human diet, however there is accumulating evidence on the benefits of polyphenols in non-communicable diseases such as cancer, cardiovascular disease (CVD) and neurodegenerative diseases. Polyphenols are divided into classes based on their structure and the number of aromatic rings they contain. The polyphenol structure consists of two or more six-carbon aromatic rings with several hydroxyl groups. Phenolic acids are molecules with only one aromatic ring and as such are not true polyphenols but are still considered within as polyphenols. The three main classes of polyphenols are flavonoids, lignans and stilbenes [62,63].

Flavonoids are the largest and most studied group of polyphenols. There are six subclasses of flavonoids commonly found in foods including, flavanols, flavanones, isoflavones, flavones, anthocyanidins, flavonols. Many flavonoids are plant pigments and are found in a wide variety of plant foods. Stilbenes are composed of two phenolic rings and are commonly found in grape skins and red currants. Lignans are small plant molecules found in seeds like pumpkin seeds, sesame seeds and flaxseeds. They are commonly confused with lignins (insoluble fiber found in plant cell walls) but are structurally very different. As opposed to lignins, lignans offer no structural support to plants. Phenolic acids, as stated above, are not true polyphenols because they only contain one phenolic ring [62,63].

Polyphenols are natural antioxidants and are hypothesized to play a beneficial role in the prevention of non-communicable diseases such as neurodegenerative disease. A systematic review evaluated studies on the effects of all polyphenols on the development of AD. Twenty-four studies were included in this systematic review, including both randomized-controlled trials and prospective observational studies. Twelve studies were found to have a positive correlation with reduced cognitive decline, 7 studies found mixed results and 5 studies found no correlation thus providing inconclusive evidence. It’s important to point out that polyphenols are a diverse group of compounds that vary greatly in molecular structure and properties. Furthering the complexity of polyphenols is their high instability during food processing and/or extraction processes. This make polyphenols difficult to study and can result in varied findings in research [64].

A recent meta-analysis analyzed randomized control trials involving people taking polyphenol-based supplements and the effects on CVD and neurodegenerative diseases. In particular, in order to assess studies on cognitive decline, this review selected studies that examined amounts of polyphenol-rich extracts up to 480 mg for Gingko biloba, 300 mg for resveratrol, and 100 mg for soy isoflavones. The findings of this meta-analysis and review were inconsistent across several studies. More research is needed to determine if single polyphenols may be of benefit in the prevention of cognitive decline. Though it is uncertain whether or not individual polyphenolic compounds produce the same beneficial results as do polyphenol rich diets [65] consumption of extracts from polyphenol sources have shown promise [66,67,68].

### 3.2. Vitamin E

One of the important phytochemicals with potent antioxidant activity is vitamin E [32]. Vitamin E is a lipid soluble antioxidant essential for normal neurological functioning composed of 4 tocopherols and 4 tocotrienols [69]. Because of its antioxidative properties, it has been reported to assist in the prevention of AD [70]. Alpha tocopherol is the most bioavailable and thus most abundant form in humans and reduced levels have been found in subjects with AD, MCI and poor memory [71]. Though alpha tocopherol is the most researched, all of the vitamin E forms show antioxidant activity and possess unique properties and modulate different signaling pathways [69]. A cross sectional, multicenter study assessed the plasma vitamin E levels and markers of antioxidative and nitrosoactive damage in 168 AD patients, 166 MCI patients and 187 cognitively normal people. They determined that compared with cognitively normal subjects, AD and MCI patients had lower levels of total tocopherols, total tocotrienols, and total vitamin E. Furthermore, a multivariable-polytomous-logistic regression analysis indicated that low plasma tocopherols and tocotrienols levels are associated with increased odds of MCI and AD [72].

In pre-clinical studies, tocotrienols have been shown to have neuroprotective properties, particularly of brain white matter, independent of their antioxidant properties [73]. White matter lesions (WMLs) are abnormal regions in the brain reported to be a major risk factor for stroke, a cause of loss of function in cerebrovascular disease and are associated with cognitive impairment. WML progression has been advocated as a marker to evaluate the efficacy of various interventions in research and clinical scenarios [74,75]. In a clinical trial 121 subjects with magnetic resonance imaging (MRI) confirmed WMLs were randomly assigned to receive 200 mg mixed tocotrienols or placebo twice a day for 2 years; 88 finished the study. The mean WML volume of the placebo group increased after 2 years, whereas that of the tocotrienol-supplemented group was unchanged. The mean WML volume change between the 2 groups was not significantly different at the end of 1 year but was significant at the end of 2 years. The authors concluded that mixed tocotrienols attenuated the progression of WMLs [76].

## 4. Palm Fruit Bioactive Complex

### 4.1. Chemistry and Mechanism of Action

PFBc is derived from the extraction process of palm oil from oil palm fruit (*Elaeis guineensis*) utilizing a patented (US patent no 7387802) process [77] that yields the water soluble phytochemical rich liquid rich in 3 isomers of caffeoylshikimic acid, 4-hydroxybenzoate, protocathechuic acid and caffeic acids as well as catechins, soluble fibers, and shikimic acid. PFBc contains 5 unique polyphenols, 3-O-caffeoylshikimic acid, 4-O-caffeoylshikimic acid, 5-O-caffeoylshikimic acid, p-hydroxybenzoic acid, and protocatechuic acid [78]. Phenolic compounds occur in many plants and portray a variety of bioactive properties mostly due to their action as an antioxidant [32]. Their antioxidant activity is influenced by position and degree of hydroxylation, polarity, solubility, reducing potential, and stability of the phenoxy radical. Crude and ethanol extracts of oil palm fruits have been shown to scavenge free radicals by either hydrogen or electron donating mechanisms [32]. In addition to functioning as antioxidants, phenolics are also known to influence gene expression [40]. Specifically, polyphenolic compounds have been found to impact the expression of COX-2 [41] through suppression of activation of nuclear factor AP-1. In addition, they have been shown to increase the expression of serum high density lipoprotein-associated paraoxonase 1 (PON-1) [43] and of antioxidant enzymes [79], to downregulate Vascular endothelial growth factor (VEGF) expression in human aortic endothelial cells [80], interferongamma (IFN-γ) and interleukin-4 (IL-4) gene expression in normal peripheral blood mononuclear [81] and in macrophages [82].

### 4.2. Research

#### 4.2.1. Pre-Clinical

The crude and ethanol extracts of palm fruits have demonstrated radical scavenging activity and exhibited hydrogen-donating capacity and have antiradical power (ARP) comparable to ascorbic acid and can therefore act as antioxidants [32]. In vitro and animal studies support the health benefits of the phytochemical PFBc, also known as oil palm phenolics (OPP), and palm fruit juice (PFJ). OPP intake was positively correlated with an anti-hyperglycemic effect in diabetic rats suggesting it may slow the rate of glucose absorption, reduce insulin resistance and/or enhance insulin secretion. [83]. OPP has been shown to improve cognitive function and spatial learning in mice [84]. OPP has demonstrated anti-tumor effects in human pancreatic cancer cells by acting on the nuclear factor-ĸB (NFĸB) signaling pathway, a ubiquitously expressed transcription factor that is responsible for stimulating inflammatory responses due to viral, hormonal, physical and oxidative stressors [85]. OPP has also been shown to lower the immune response of tumor bearing mice compared to controls, suggesting delayed inflammation in OPP supplemented mice [86]. PFJ has been shown to offset oxidative stress and 3′-Azido-3′-deoxythymidine (AZT)-induced mitochondrial genotoxicity (mutagenesis) and dose-dependent cytotoxicity in HepG2 (a human liver cancer cell) cells suggesting that PFJ exerts a protective effect on the mitochondria [87]. This may be of particular importance for age related changes associated with inflammaging which have been associated with increases in mitochondrial prooxidants. In an in vitro study OPP was shown to inhibit the aggregation of amyloid beta (Aβ) into oligomers and to significantly decrease the cytotoxicity of aggregating neuropeptides in yeast genetically engineered to overexpress these peptides. This suggests the potential of OPP to reduce neuroinflammation and neuronal death associated with MCI and AD [88]. For a full review of potential mechanisms of OPP on neuroprotective actions see Ibrahim et al. (2020).

While oxidation and inflammation are associated with aging, it has been established that aging is regulated by genes that are similar across species [89]. In addition to their antioxidant activities, phytochemicals such as phenolics have been shown to influence gene expression [40]. In vivo OPP has shown various pleiotropic effects in normal mice including up regulation of four lipid catabolism genes and down regulation of five cholesterol biosynthesis genes in livers, up regulation of eighteen blood coagulation genes in spleens and gene expression changes similar to the effects of caloric restriction in the hearts of mice supplemented with OPP. The authors concluded that OPP may play a role in reducing CVD via these mechanisms [90]. A later study further supported the role of OPP in potentially reducing risk of atherosclerosis and CVD by feeding mice an atherogenic diet. Supplementation with OPP attenuated the oxidative stress caused by the diet. Unfolded protein response in the liver was increased, antigen presentation and processing in the spleen was attenuated, and antioxidant genes in the heart were up regulated. OPP attenuated inflammation by modulating the type 1 T helper/type 2 T helper (Th1/Th2) axis toward the latter. OPP also increased serum antioxidant activity to normal levels [91].

OPP given to mice up-regulated genes involved in brain development under the regulation of brain-derived neurotrophic factor (BDNF) and down regulated genes related to inflammation suggesting a mechanism by which OPP may improve memory and reduce cognitive impairment [84].

Genes that regulate aging involve signaling related to DNA damage response, mitogen-activated, nutrient-sensing and stress-responsive pathways [92]. Phytochemicals may contribute to longevity via these pathways and thus enhance survival much the way they do in plants. In plants, phytochemicals help the plant to survive various stressors such as ultraviolet (UV) light, nutrient deprivation, etc. improving longevity. Phytochemicals can extend longevity in heterotrophic organisms across phyla via evolutionarily conserved mechanisms by modulating a network of signaling pathways that influence longevity via cellular processes [93]. Whether this extends to other organisms is not clear but is tested via longevity regulating mechanisms in naturally short-lived organisms such as yeast and fruit fly. Microarray gene expression analysis was performed on whole fruit fly larvae and their fat bodies, after the larvae were fed a control Standard Brandeis Diet (SBD) with or without PFJ. Eclosed fruit flies fed PFJ or its fractions during the larval stage, then aged on PFJ-free SBD showed 20–40% improved survival ratings over controls. Transcriptomic analysis on whole fruit fly larvae and larval fat bodies revealed that PFJ supplementation generally lengthened the active stage of the fruit fly larvae and this may thus delay their aging process via expression regulation of hormetic stress response genes (Tor, various heat shock proteins, and Sod2) linked to aging and longevity [94].

A diet supplemented with 10% PFBc significantly increased the level of tyrosine hydroxylase (TH) in the basal ganglia of Nile rats [95]. TH is a key and rate-limiting enzyme in catecholamine biosynthesis; it catalyzes the hydroxylation of L-tyrosine to L- Dihydroxyphenylalanine (L-DOPA) in the brain and adrenal medulla. Neurodegeneration of dopamine and noradrenaline neurons leads to deficiencies in the brain stem associated with symptoms of individuals with Parkinson’s disease (PD) [96]. Whether this increase in TH translates directly to increases in dopamine, norepinephrine and epinephrine in the rat or human brain is not clear but offers a promising mechanism of action by which PFBc may help prevent neurodegenerative disease. Further research in this area is warranted.

#### 4.2.2. Human Clinical Trials

While pre-clinical studies assessing gene expression and beneficial effects of PFBc are promising, it is important to determine benefits in human clinical trials. In a pilot, exploratory randomized, double-blind, placebo-controlled study, the effects of three doses of PFBc (0 mg/Placebo, 250 mg, 500 mg and 1000 mg/day) taken for 30 days in 13 male and 16 female healthy physically active subjects on measures of physiology, mood state, cellular damage, and gut microbiome status was evaluated. PFBc enhanced antioxidant capacity (cORP) at rest for all three doses tested while there was no effect of placebo. In addition two doses of the PFBc trended towards enhancing antioxidant capacity after stressful exercise (500 mg from 1.1 to 2.8 (*p* = 0.64) and 1000 mg 1.67 to 2.91 (*p* = 0.058), while there was no effect for placebo (*p* = 0.320) or for the 250 mg PFBc (*p* = 0.928) [97]. This study served to demonstrate the in vivo antioxidant effects in physically active individuals, which is opening up greater research interest. This is supported by a study by Ojeda et al. that showed that supplementation of 25 mL of *Elaeis oleifera* × *Elaeis guineensis* hybrid palm oil (HPO) for a period of 3 months significantly increased (*p* < 0.01) the total phenolic content (19.2%) as well as the antioxidant capacity of human plasma measured by both Oxygen Radical Absorbance Capacity ORAC (92.1%) and Trolox equivalent antioxidant capacity (TEAC) (42.9%) methods in healthy subjects over 50 years of age [98].

A study was conducted to assess the safety and efficacy of *Elaeis guineensis* and *Ficus deltoidea* leaf extracts in adults with pre-diabetes. Subjects were randomly assigned to receive *E. guineensis* leaf extract 500 mg or 1000 mg or *F. deltoidea* leaf extract 1000 mg. *E. guineensis* intervention for 8 weeks decreased fasting plasma glucose and insulin levels, glucose and insulin areas under the curve, and insulin resistance, and increased insulin sensitivity. The 500 mg dose of *E. guineensis* had a more consistent effect on reducing glycemia than the 1000 mg dose and the insulin findings at the two dose levels were somewhat inconsistent. There were no issues with safety [99].

Thirty healthy subjects were randomly assigned to receive (Elaeis guineensis) leaf alcohol extract (OPLE) (500 mg/day for 60 days) and their cognitive learning abilities were compared to placebo-controlled groups (N = 15) using a validated researcher developed test. Subjects consuming the OPLE had significantly improved scores on short term memory after 1 month that lasted for 2 months. The spatial visualisation ability and processing speed improved (*p* < 0.05) after 2 months consumption [100].

#### 4.2.3. Safety

PFBc has been determined to be safe by an independent Generally Recognized as Safe (GRAS) panel. The GRAS Panel was selected and convened in accordance with the United States (U.S.) Food and Drug Administration (FDA)’s guidance for industry on Best Practices for Convening a GRAS Panel [101]. PFBc has been shown to benefit several diseases in animal models with no evidence of toxicity [77,83,86,90,91,102,103,104]. It has been demonstrated that PFBc was safe and well-tolerated in healthy human volunteers at doses up to 1000 mg/day for 30 days [97]. A 90-day sub-chronic toxicity study in Sprague Dawley rats was done to assess safety of water-soluble palm fruit bioactives in food. No significant effects were noted on body weight, food consumption, hematology, clinical chemistry, organ weights, and histopathological examination. The No Observable Adverse Effect Level was considered to be 2000 mg/kg body weight/day, the highest dose tested [78]. A phase one clinical trial was conducted to evaluate the safety and effects of OPP in 25 healthy volunteers over 60 days. Subjects were given 450 mg gallic acid equivalent (GAE)/day (equivalent to 12,000 mg PFBc/day) of OPP or control treatments. There were no adverse events, and the subjects experienced an improvement in total cholesterol and low-density lipoprotein cholesterol (LDL-c) levels compared to controls [105].

## 5. Conclusions

As the number of aged individuals increases worldwide there is interest in defining and achieving healthy aging. As a critical part of achieving healthy aging is mental and cognitive health, determining mechanisms for preventing and mitigating MCI is of critical importance. While not everyone with MCI develops AD, preventing one potentially prevents the other. The aging process is associated with activation of inflammatory processes called inflammaging. In turn, the production of prooxidants via oxidative stress from mitochondrial damage further exacerbates the inflammation. Prooxidants play an important role in gene expression, and their role in inflammaging is thought to occur through their impact on transcription factors. Therefore, an increase in antioxidants via diet or supplementation has been suggested. This makes sense when one considers that antioxidants including phenolics influence gene expression. Therefore, a diet or supplemental protocol high in antioxidants, particularly phenolics may help to mitigate chronic disease by decreasing inflammation and may do so at the genetic level. The Mediterranean Diet and related dietary patterns have been suggested to provide phenolics, and supplemental PFBc has also been suggested as a phenolic that may offset inflammaging by influencing gene expression. Several in vitro, in vivo and animal studies support multiple benefits of PFBc especially for improving cognitive function via anti-inflammatory and antioxidant mechanisms. While more human studies are needed, those completed thus far support the potential benefit of consuming PFBc to enhance cognitive function via its anti-inflammatory antioxidant functions.

## Data Availability

Data sharing not applicable. No new data were created or analyzed in this study. Data sharing is not applicable to this article.
